# Measuring HIV outcomes for adolescent girls and young women programs in Africa: Using the polling booth survey technique

**DOI:** 10.1371/journal.pone.0307198

**Published:** 2024-07-22

**Authors:** Faran Emmanuel, Lize Aloo, Amna Mahfooz, Mathato Nkuatsana, Nametsego Tswetla, Nicolus Mutenda, Biziwick Mwale, Zounkanyi Bissek, Parinita Bhattacharjee

**Affiliations:** 1 Institute for Global Public Health, University of Manitoba, Winnipeg, Canada; 2 The Global Fund to Fight AIDS, TB and Malaria, Geneva, Switzerland; 3 Centre for Global Public Health, Islamabad, Pakistan; 4 PACT, Maseru, Lesotho; 5 Ministry of Health and Wellness, Gaborone, Botswana; 6 Ministry of Health & Social Services, Windhoek, Namibia; 7 World Vision International, Lilongwe, Malawi; 8 Cameroon National Planning Association for Family Welfare (CAMNAFAW), Bamenda, Cameroon; 9 Partners for Health and Development in Africa, Nairobi, Kenya; United States Agency for International Development (USAID), NIGERIA

## Abstract

**Introduction:**

Adolescent girls and young women (AGYW) remain highly vulnerable to the risk of acquiring HIV (Human immunodeficiency virus). This study was conducted to measure behavioral, biomedical and structural outcomes for the Global Fund funded AGYW programmes in five African countries with high burden of HIV including Botswana, Cameroon. Lesotho, Malawi and Namibia.

**Methods:**

The study used a mixed methods approach to collect behavioral, structural and biomedical outcome data. Quantitative data were collected through 418 Polling Booth Survey (PBS) sessions from 4,581 AGYWs. Participants were recruited through a community-based multistage sampling technique using sampling weights for urban and rural communities. 23 Focus Group Discussions (FGD) were conducted to understand barriers to use of HIV prevention programme and community recommendations for improved coverage. Ethical approvals were obtained from the ethics review board in all five countries.

**Results:**

More than 50% of the respondents from all five countries reported to be sexually active, and at least 30% or more of those who were sexually active had multiple sex partners. There were wide variations between the countries in condom use with a non-marital sexual partner which ranged between 66% in Namibia to 42% in Cameroon. Cameroon (44%) had high percentage of AGYWs with independent income source while school drop-outs were higher in Malawi (55.5%) and Lesotho (46.6%). Nearly 1/4th of AGYWs in all countries, except Namibia, reported experiencing intimate partner violence. Nineteen percent of the respondents were pregnant in the last 12 months, and 50% of those pregnancies were unplanned. Lesotho had the highest proportion of AGYW (90.5%) ever tested for HIV, followed by Malawi (87.5%), Botswana (75%), Cameroon (69%) and Namibia (62.6%).

**Discussion:**

There is diversity across the countries, with country-wise and age-wise variations in results. In all countries, the AGYW programme will benefit from a more targeted approach to reach out to the most vulnerable AGYW, strengthening structural interventions, strengthening linkage to PrEP (Pre-Exposure Prophylaxis) and ART (Antiretroviral Therapy) for those who are living with HIV and a strong linkage with reproductive health services. The assessment helped countries to understand the gaps and opportunities to improve the HIV prevention programme with AGYW.

## Introduction

Adolescents and young people represent a growing share of people living with HIV worldwide [1]. While HIV incidence has shown a declining trend over the last decade among general population across Africa, the number of new HIV infections among adolescents is on a rise [2]. In 2022, in sub-Saharan Africa, women and girls (of all ages) accounted for 63% of all new HIV infections. Adolescent girls and young women accounted for more than 77% of new infections among young people aged 15–24 years in 2022 in sub-Saharan Africa [3]. It is also estimated that every week in 2022, nearly 4,000 adolescent girls and young women (AGYW), became infected with HIV; 3,100 of these infections occurred in sub-Saharan Africa [3]. In some sub-Saharan countries, adolescent girls are two to three times more likely to be infected with HIV than boys of the same age group [4,5].

A number of biological, structural and behavioral factors drive the proliferating growth of HIV among AGYW and puts them at the epicenter of the HIV epidemic in many African contexts. They are highly susceptible to HIV infection due to an immature genital tract and a greater proportion of genital mucosa exposed to HIV [6,7]. The risk is compounded by other contextual factors such as initiation of sex at a younger age, high prevalence of sexually transmitted infections (STIs) and non-availability of condoms or inability to negotiate safe sex [8–11]. One of the key factors is their engagement in sexual relationships with older men with unequal power relationships and are often not able to negotiate for safer sex or other preventive practices [12,13]. To fill these prevention gaps, there is a need to reach out to AGYW for provision of program services as shown by the results of Evidence for Contraceptive Options in HIV Outcomes study (ECHO), which revealed a critical gap in integrated HIV prevention and SRH services targeting the AGYW [14] Click or tap here to enter text.

According to the global AIDS strategy 2021–2026, it is important for countries to invest in new generations of youth leadership to ensure the sustainability of the HIV response [15]. Conforming to this, the Global Fund aims to protect and promote human rights and gender equality through “scaling up programmes to support women and girls” using an evidence-informed package of interventions for HIV prevention and treatment for AGYW in 13 of the countries with highest HIV burden [16]. Most AGYW programs are faced with the challenge of generating granular outcome data on a timely basis to demonstrate progress towards desired impact [17]. We therefore conducted this study in five selected countries with the purpose to gather sexual, behavioral, biomedical and structural outcomes of the AGYW program using a simple and a cost effective approach that could be effectively used by the programs to periodically collect and analyze outcome data for more dynamic decision-making [18].

## Methodology

### Study design and settings

We used a mixed-method design including both quantitative and qualitative approaches. Quantitative data was collected through a cross sectional survey using Polling Booth survey (PBS) approach, which is a rapid, simple group interview method and individuals give their responses through a ballot box in an individual polling booth [19]. In addition, focus group discussions were conducted to collect qualitative data. The study was implemented across five countries including Botswana, Cameroon, Lesotho, Malawi, and Namibia and was limited to sub- national geographic areas where the Global Fund supported AGYW programs are implemented. The overall research strategy and implementation approach was developed by the technical team including the University of Manitoba’s research team, the GFATM country and global AGYW leads and the technical staff of AGYW programs in the countries.

### Sample size and Sampling distribution

Sample size was calculated to detect a 10% change in the primary outcomes between this survey and a follow-up end line survey with 95% statistical confidence and 90% statistical power. A design effect of 2 was added and the sample was inflated by 5% to adjust for data errors and non-response. The calculated sample size of 1,080 was adjusted for each country using the finite population correction factor. The sample was allocated proportionally to each district, based on the distribution of younger (15 to 17 years) and older (18 to 24 years) AGYW (see Table 1).

**Table 1:** Sampling distribution of AGYWs within the targeted countries.

Country	Sample Size	Sample distribution				No of PBS	No of FGDs
		District	Total AGYW	Younger AGYW	Older AGYW		
				(15 to 17)	(18 to 24)		
Cameroon	996	Nkolondongo	276	120	156	69	4
		Bertoua	252	108	144		
		Dcshang	108	48	60		
		Bonassama	180	48	132		
		Garaoua	180	60	120		
Malawi	1056	Mulanje	192	80	112	88	5
		Lilongwe	60	24	36		
		Thyolo	180	72	108		
		Mangochi	624	192	432		
Botswana	808	Francistown	164	20	144	90	5
		Maun	140	24	116		
		Palapye	168	24	144		
		Selebi Phikwe	168	36	132		
		Tutume	168	24	144		
Lesotho	1020	Botha Bothe	181	70	111	83	5
		Leribe	379	137	242		
		Mokhotlong	177	69	108		
		Qacha’s Nek	109	56	54		
		Thaba Tseka	173	72	101		
Namibia	1020	Ohangwena	526	242	284	88	4
		Omusati	494	142	352		

Eligible AGYW were selected randomly from the sampled urban and rural areas, following a multistage probability sampling technique. Firstly, within each country, 5 districts were randomly selected from the total number of districts where the intervention was implemented. In Malawi and Botswana, the program was implemented in fewer than five districts and therefore all intervention districts were selected. The next step involved selection of villages and towns, for which a list of all urban neighborhoods and villages, along with an approximate number of AGYW (stratified by age) was obtained. Sampling weights, based on the number of AGYW were applied and a random sample of villages and towns was selected. In the final stage, we randomly selected respondents from households, based on age stratification i.e., AGYW aged between 15–17 years or 18–24 years. In Cameroon the younger age group included girls up to 20 years old, based on country’s definition of younger and older AGYWs. If more than one eligible girl resided in the house, a random selection of one respondent was done from the household. Recruitment of eligible participants was done by peer educators along with the research coordinator.

### Data collection processes and field teams

Data were collected within 30–40 days between April to November 2021 in Botswana, Lesotho, Malawi and Namibia, and between July to September 2022 in Cameroon. Each country had a different date of ethical approval followed by its data collection. Within each country, a research team including a research coordinator and 8 to 10 field workers was recruited. Since the UM technical team couldn’t travel due to COVID-19 related travel restriction, local research teams were trained virtually in a three-day training workshop by UM technical team. The research teams were further provided with video guidance on how to conduct PBS and a 24/7 helpline assistance provided by UM technical staff. Field support was provided by implementing partners, as research teams visited the selected villages and towns, and randomly selected households to recruit respondents. Potential respondents went through an eligibility screening and if found eligible, were requested to consent as per the protocol and participate in the assessment. Recruitment period and data collection was done from 9–29 July, 2021 in Malawi,14–26 April 2021 for Botswana, 8–19 November, 2021 for Namibia, 17–31 May, 2021 for Lesotho, and 29July-9Sep, 2021 for Cameroon. For girls younger than 18 yrs, a parental consent was obtained. PBS were conducted with groups of 10–12 AGYWs, stratified by age who were invited at the data collection site on specific days and times suggested during the community mobilization process. Once the girls arrived at the data collection site, her eligibility was re-checked and informed consent was confirmed. PBS is a group interview method and individuals gave their responses through a ballot box in an individual polling booth in the venue. Each participant was provided three colored boxes i.e., red, green and white, along with a pack of cards numbered corresponding to the number of questions in the questionnaire. The moderator asked questions in the local language, one at a time, and the respondents answered by putting the numbered card in one of the three boxes i.e., green box if the response is YES, red if NO, white if the question didn’t apply. The card was kept outside if the respondent didn’t want to respond to a question. Once the entire questionnaire was administered, the responses to each question were collected and tabulated on the polling booth survey tally sheet, based on the total number of cards that were found in the red, green, and white boxes.

FGD were conducted with respondents of one of the randomly selected PBS group within each district. FGDs were conducted in local language and the proceedings were recorded. Each FGD was conducted by a team of 2 researchers; the moderator led the discussions using a FGD guide, while the other researcher recorded the responses. FGDs focused on understanding the reasons behind some of the risk key outcomes, as well as the participant’s experience in accessing health services and their satisfaction with the program services. It also encouraged participants to suggest any additional services that they would like to be provided through the program.

### Data collection tools, data management and analysis

PBS used a standardized structured tool across all 5 countries which captured data on all key outcome indicators including sexual partnerships and condom use, HIV testing, treatment and care, use of PrEP, school dropout, income, intimate partner violence, gender equality and access to AGYW programme. FGDs investigated the reasons for risk key outcomes, and the participant’s experience of AGYW programs. Tally sheets from all PBS were thoroughly checked for inconsistencies, missing values and were entered in a data base designed in MS Excel. Any data entry errors were corrected, and the data file was prepared for analysis.

Frequencies were run for all variables, calculating the number of responses and percentages within each category of response for each participating country. Outcome measures were calculated for each country separately stratified by younger (15 to 17 years) and older (18 years and above) AGYWs. Data gathered from FGDs was transcribed and thematic interpretive analysis was done to draw out and explore individual and shared group meanings pertaining to the central assessment objects. The analysis involved finding themes, coding, and writing summary notes.

### Ethical considerations

This research followed all ethical principles of research including parental consent (AGYW below 18 yrs) and/or oral informed consent from all participating AGYWs were taken and form signed on behalf of the participants. Ethical approvals were obtained from an ethical review board (ERB) within each country. Ethical approvals were obtained from: 1) Ministry of health and wellness, republic of Botswana, 2) Ministry of health and social sciences, republic of Namibia 3) Ministry of health, Lesotho 4) Ministry of public health, Cameroon 5) Ministry of health and population, Malawi. The assessment reimbursed the participants for their time and travel. In this outcome assessment, the participants were given 5 USD in total to compensate for their time and travel.

## Results

A total number of 4,581 AGYW participated in 418 polling booth surveys (93.5% of the sample size calculated) conducted across all five countries. Among these, 3,053 AGYW belonged to the older age group (18 to 24 yrs) while the remaining 1,528 were between 15 to 17 yrs of age (Cameroon’s younger age group included girls up to 20 years old, based on country’s definition of younger and older AGYWs). In addition, 23 FGDs, 9 with younger AGYW and the remaining 14 with older AGYW were conducted. The nonresponse rates varied among countries, with an average response rate of nearly 95% for all countries.

### Structural outcomes

Table 2 shows assessment of various structural outcomes with wide inter-country variations. School dropout rates varied between the five countries ranging between 56% in Malawi to 16% in Namibia, with a higher school dropout rate among older AGYWs in all countries. Education was highly valued among the respondents as revealed in the qualitative findings and reasons for dropouts included poverty, culture, violence in school, unplanned pregnancies, and poor performance among others. A higher proportion of respondents in Cameroon (44%) and Malawi (35%) had a source of regular income as compared to Botswana (28.3%), Namibia (16%) and Lesotho (11%), higher among older AGYW in comparison to younger AGYWs. Higher number of AGYW reported experiencing gender discrimination by family members than from the community. Respondents from Malawi reported highest level of discrimination both by the family and community at 30% and 26% respectively, followed by Botswana (28% and 18%) and Cameroon (23% and 25%). Overall, older girls reported higher experience of gender-based discrimination within the family as well as the community. Intimate partner violence (IPV) was reported by more than half of the AGYWs, with higher level of physical violence than sexual violence. No age-specific trends were noticed and both groups faced violence (physical and sexual) from their intimate partners indistinctly. Girls also discussed violence as one of their key issues in FGDs. They reported emotional abuse from intimate partners like insults and nags, financial abuse e.g. taking away earnings and sexual abuse like forced sex by boyfriends, husbands and cohabiting partners. Reporting of violence is less as there is fear of relationship break up, therefore they mentioned reporting intimate partner violence only in extreme cases.

**Table 2:** Structural outcomes among AGYWs in 05 African countries.

	Age Stratification	Botswana n (%)	Lesotho n (%)	Malawi n (%)	Namibia n (%)	Cameroon n (%)
Dropped out of school	**Overall**	**142 (18)**	**403 (47)**	**557 (56)**	**162 (16)**	**258 (36)**
	Young AGYW	20 (16)	116 (35)	158 (45)	55 (13)	87 (35)
	Older AGYW	122 (19)	287 (54)	399 (61)	107 (18)	171 (37)
Independent source of income	**Overall**	**226 (28.3)**	**99 (11)**	**360 (35)**	**146 (16)**	**345 (44)**
	Young AGYW	19 (15.2)	35 (10)	105 (29)	49 (13)	103 (39)
	Older AGYW	207 (30.8)	64 (12)	255 (38)	97 (18)	242 (47)
Intimate partner violence (Physical)	**Overall**	**100 (78)**	**70 (60)**	**67 (55)**	**45 (58)**	**62 (55)**
	Young AGYW	10 (91)	19 (54)	14 (54)	17 (68)	14 (37)
	Older AGYW	90 (77)	51 (63)	53 (55)	28 (54)	48 (65)
Intimate partner violence (Sexual)	**Overall**	**63 (49)**	**64 (55)**	**73 (60)**	**45 (58)**	**52 (46)**
	Young AGYW	8 (73)	20 (57)	15 (58)	14 (56)	12 (32)
	Older AGYW	55 (47)	44 (54)	58 (60)	31 (60)	40 (54)
Experienced gender-based discrimination (family)	**Overall**	**225 (28)**	**142 (17)**	**315 (30)**	**156 (17)**	**163 (23)**
	Young AGYW	25 (19)	54 (16)	106 (29)	70 (18)	46 (19)
	Older AGYW	200 (30)	88 (17)	209 (31)	86 (16)	117 (25)
Experienced gender-based discrimination (community)	**Overall**	**146 (18)**	**100 (12)**	**272 (26)**	**145 (16)**	**178 (25)**
	Young AGYW	16 (12)	35 (11)	87 (24)	55 (14)	48 (20)
	Older AGYW	130 (19)	65 (12)	185 (28)	90 (17)	13 (07)

### Behavioral outcomes

Table 3 presents various behavioral outcomes assessed, which shows that more than half of the girls in all five countries reported of being sexually active (ranging between 74% in Botswana to 54% in Namibia). Among those who were sexually active in the last 12 months, at least one-third reported having sex with multiple partners; 42% highest in Botswana, followed by Cameroon (39%), Malawi (38%), Namibia (34%) and Lesotho (30%). Surprisingly, higher proportions of younger AGYW in Namibia (59% younger vs 28% older girls) and Lesotho (37% younger vs 28% older girls) reported multiple sex partners in comparison to older girls. Condom use at last sex was reported by more than half of the sexually active AGYWs: higher condom use was reported by younger AGYWs across all countries except Namibia. Having a non-marital non-cohabiting partner (NMNC) ranged between 47% in Cameroon to 13% in Namibia. Condom use with NMNC partners showed a significant age-related stratified difference, as older girls reported higher condom use in comparison to younger girls except in Cameroon (45% younger vs 41% older girls). FGDs informed that dating older men who have steady work for financial benefits is common in all countries. Lack of parental guidance, peer pressure to fit in within the social circle and influence of social media are some of the major reasons for engaging in sexual relationships. Condom use is low as AGYWs feel shy to collect condoms from hospitals or public places and are also scared to ask their steady partners to use a condom because of the fear of breaking trust. Non availability of condoms is another major reason for its inconsistent use as mentioned in the FGDs.

**Table 3:** Behavioral outcomes among AGYWs in 05 African countries.

	Age Stratification	Botswana n (%)	Lesotho n (%)	Malawi n (%)	Namibia n (%)	Cameroon n (%)
**Ever had sex**	**Overall**	**602 (74)**	**581 (68)**	**738 (71)**	**509 (54)**	**416 (55)**
	Young AGYW	46 (35)	159 (48)	171 (47)	139 (36)	129 (52)
	Older AGYW	556 (82)	422 (80)	567 (84)	370 (67)	287 (57)
**Multiple sex partners (last 12 months)**	**Overall**	**233 (42)**	**143 (30)**	**184 (38)**	**106 (34)**	**126 (39)**
	Young AGYW	14 (37)	46 (37)	39 (38)	37 (59)	30 (29)
	Older AGYW	219 (42)	97 (28)	145 (38)	69 (28)	96 (43)
**Condom use at last sex**	**Overall**	**385 (64)**	**378 (65)**	**410 (56)**	**359 (71)**	**229 (55)**
	Young AGYW	34 (74)	127 (80)	102 (60)	95 (68)	73 (57)
	Older AGYW	351 (63)	251 (59)	308 (54)	264 (71)	156 (54)
**Has a non-marital non-cohabiting partner (NMNC)**	**Overall**	**300 (38)**	**131 (16)**	**280 (27)**	**90 (13)**	**318 (47)**
	Young AGYW	30 (23)	45 (14)	64 (18)	49 (18)	114 (50)
	Older AGYW	270 (41)	86 (17)	216 (32)	41 (9)	204 (45)
**Condom use at last sex with NMNC partner**	**Overall**	**189 (63)**	**73 (56)**	**168 (60)**	**59 (66)**	**134 (42)**
	Young AGYW	18 (60)	18 (40)	38 (59)	23 (47)	51 (45)
	Older AGYW	171 (63)	55 (64)	130 (60)	36 (88)	83 (41)

### Biomedical outcomes

Table 4 shows that Lesotho had the highest proportion of AGYW (90%) ever tested for HIV, followed by Malawi (88%), Botswana (82%), Cameroon (69%) and Namibia (63%). A high proportion of AGYW self-reported living with HIV. Botswana had the highest proportion of AGYW (21%) living with HIV, followed by Lesotho (12%), Cameroon (10%), Namibia (9%) and Malawi (5%). On average only 55% of AGYW living with HIV were taking antiretroviral therapy. Low Use of PrEP among AGYW across countries was reported. Less than 10% of AGYW reported ever taking PrEP in all countries except for Cameroon, where it was 30%. Among those who had ever taken PrEP, a high percentage of the respondents in Botswana (78%) and Namibia (68%) were currently taking PrEP compared to Cameroon (39%), Malawi (35%) and Lesotho (25%). Higher proportion of younger AGYW were currently taking PrEP compared to older AGYW except in Namibia.

**Table 4:** Biomedical and SRH related outcomes among AGYWs in 05 African countries.

	Age Stratification	Botswana n (%)	Lesotho n (%)	Malawi n (%)	Namibia n (%)	Cameroon n (%)
**ever tested for HIV**	**Overall**	**593 (75)**	**788 (90)**	**914 (88)**	**595 (63)**	**535 (69)**
	Young AGYW	60 (48)	297 (88)	290 (79)	183 (47)	177 (69)
	Older AGYW	533 (80)	491 (92)	624 (92)	412 (74)	358 (69)
**Living with HIV**	**Overall**	**142 (21)**	**96 (12)**	**40 (5)**	**59 (9)**	**58 (10)**
	Young AGYW	15 (15)	38 (13)	13 (5)	21 (10)	8 (4)
	Older AGYW	127 (22)	58 (12)	27 (5)	38 (9)	50 (13)
**Currently taking ART**	**Overall**	**105 (74)**	**48 (50)**	**22 (55)**	**34 (58)**	**22 (38)**
	Young AGYW	9 (60)	13 (34)	4 (31)	8 (38)	5 (63)
	Older AGYW	96 (76)	35 (60)	18 (67)	26 (68)	17 (34)
**Ever taken PrEP**	**Overall**	**63 (8)**	**40 (5)**	**40 (4)**	**41 (5)**	**197 (30)**
	Young AGYW	8 (6)	7 (2)	9 (2)	19 (5)	62 (27)
	Older AGYW	55 (8)	33 (7)	31 (5)	22 (5)	135 (31)
**Currently taking PrEP**	**Overall**	**49 (78)**	**10 (25)**	**14 (35)**	**28 (68)**	**77 (39)**
	Young AGYW	7 (88)	3 (43)	3 (33)	6 (32)	27 (44)
	Older AGYW	42 (76)	7 (21)	11 (35)	22 (100)	50 (37)
**Have you been pregnant? (last 12 months)**	**Overall**	**185 (23)**	**143 (17)**	**269 (26)**	**141 (16)**	**105 (15)**
	Young AGYW	18 (15)	17 (5)	60 (17)	32 (8)	26 (11)
	Older AGYW	167 (25)	126 (24)	209 (31)	109 (22)	79 (17)
**Planned pregnancy**	**Overall**	**105 (57)**	**90 (63)**	**120 (45)**	**37 (26)**	**60 (57)**
	Young AGYW	11 (61)	6 (35)	18 (30)	9 (28)	17 (65)
	Older AGYW	94 (56)	84 (67)	102 (49)	28 (26)	43 (54)
**ever had an abortion**	**Overall**	**79 (24)**	**17 (6)**	**61 (11)**	**54 (26)**	**101 (47)**
	Young AGYW	16 (76)	3 (8)	11 (11)	17 (44)	27 (43)
	Older AGYW	63 (20)	14 (6)	50 (11)	37 (22)	74 (48)

We also inquired about other sexual and reproductive health (SRH) indicators including unwanted pregnancies and abortions. Among those who were pregnant in the last 12 months, nearly two-third pregnancies among young AGYWs were unplanned in Lesotho, Namibia and Malawi. Among those who were ever pregnant, Cameroon had a high proportion of girls (47%) who had an abortion, followed by Namibia (26%), Botswana (24%), Malawi (11%) and Lesotho (6%). Qualitative discussions informed that pregnancy is a real concern as they face judgement from the society and rejection from the family and therefore prefer to get it terminated. Lack of availability of family planning pills and various SRH services was one of the serious concerns raised in FGDs.

## Discussion

This assessment has improved our understanding of the sexual, behavioral, biomedical and structural factors associated with HIV-related risk and vulnerability among AGYW in five African countries. Our results reveal a wide array of vulnerabilities which lead to a high prevalence of risk behaviors including early sexual activity, multiple sex partners and unprotected sex. Our findings have also highlighted various structural factors which might play a role in driving behaviors, that increase the likelihood of HIV and STI transmission. The results show substantial heterogeneity between countries and across younger and older adolescents, and the prevention response needs to be adapted to these regional and age-specific diversities for an effective impact.

One of the key findings is the high prevalence of risky sexual behaviors and practices of AGYWs across all five countries among both younger and older girls. Risky sexual behaviors can take many forms such as having multiple sexual partners and/or engaging in unprotected sex [20]. Our results are in concurrence with findings from multiple large surveys from Africa and shows that more than half of the AGYWs are sexually active, have multiple sex partners and use condoms infrequently [10,11,21]. FGDs revealed that AGYWs having multiple sexual relationships, including steady intimate partnership based on love as well as having sexual relationships with older wealthy men for financial gains and everyday life needs. Age-disparate sexual relationships with older men are a common driver of the HIV epidemic in the region [22,23]. This is largely because of unequal power dynamics in these relationships, as younger female partners of older affluent males are often treated as submissive objects and frequently exposed to gender-based violence [24,25]. Since adolescent girls develop such relationships based on economic needs, they are not in a position to negotiate safe sex as well and hence are exposed to a high risk of HIV. Our study also found inconsistent condom use across all countries which is in agreement to the reported pooled prevalence of 59.8% for inconsistent condom use in Sub Saharan Africa among adolescent girls living with HIV/AIDS [26].

While sexual behaviors are the driving force behind the spread of HIV epidemic in Africa, pragmatic research shows that adolescents’ vulnerabilities and risk practices are determined by various structural factors [27]. Our study has also looked at various structural outcomes such as educational attainment, gender inequalities, and access to services to get a more wholistic picture of the program response. School dropout varied between countries (between 16% to 56%) mostly because of an inability to pay school fees and poor performance as informed by the girls participating in FGDs. While the evidence on the association between educational attainment and HIV status across sub-Saharan Africa is not straight forward, data is suggestive that AGYWs who drop out of school are at a higher risk of being HIV positive in some sub-Saharan African settings [28]. Our results also show that most girls interviewed were financially independent. This is an important finding as programs need to include activities which lead to economic empowerment of AGYWs and reduce their engagement in transactional and age-disparate sexual relationships [29].

Investigation into some of the biomedical outcomes i.e., HIV testing and ART uptake showed that all countries fall significantly short of the 95-95-95 targets with low rates of HIV testing and low levels of adherence to ART, similar to shown by surveys from Sub-Saharan Africa [30]. Other than individual level barriers such as age, place of residence etc., mentioned by girls in FGDs, various structural barriers such as the age of informed consent, accessibility to services and stigma associated with a positive HIV test could be the limiting factor to accessing HIV testing services [30]. This slow progress in all these biomedical outcomes has severe implications for the spread of HIV and the control, cost of treatment and HIV related-deaths. However, these findings are extremely supportive of the need for continued emphasis on structural interventions directed towards reducing stigma for enhancing the effectiveness of core HIV interventions to reduce HIV incidence [31]. Another concern identified was the high number of unwanted pregnancies and abortion rates, which was also mentioned by girls participating in FGDs. The girls informed that SRH services although needed by AGYWs are not an integral part of the existing programs thus highlighting unmet contraception needs among this group and non-satisfaction with program services. AGYW programs in Africa should look into effective integration of HIV and SRH services to provide access to effective forms of modern contraception and safe abortion services for promotion of SRH rights of these girls. It would not only require providing awareness to the AGYW community but will also need focused efforts towards behavioral change interventions for health care providers [32].

IPV is crucial to the HIV epidemic among women globally and particularly in sub-Saharan Africa [33–35]. Our study revealed a high level of IPV among the respondents, but due to the design we were unable to determine the associated factors Further research is needed to elucidate the association between IPV and HIV among AGYW in these countries as well as other geographical regions, with AGYWs of diverse backgrounds and varying levels of HIV risks. The findings of this study are in accordance with available literature from Africa, which shows that low usage and demand as well as sub-optimal adherence of PrEP among AGYW [36–38]. Varying approaches to improve the access, uptake, adherence and continued use for AGYW such as PrEP delivery models, peer support, and using ethnographic and qualitative data to customize communication strategies should be tested [39–41].

A few limitations of the study need to be mentioned. A few of these limitations are inherent to the methodology of polling booth surveys. For example, being a group interview method, the responses are not traceable back to the individual and linked analysis cannot be done. Further to this, responses to PBS questions are dichotomized into a yes or no answer only. Other limitations of this study are related to the time period when data were collected. Since it was the time of COVID-19, a number of unusual delays in field were encountered and the unpredictability of the situation led to non-response rate of above 5% and an inability to achieve 100% sample size.

Despite these limitations, the study has several implications for programmes. The study shows that in all the countries, AGYW are not a homogeneous group, and have different risks and vulnerabilities to HIV and require programmes that target and focus these vulnerabilities with greater intensity. Within a particular geography, risk and vulnerabilities also vary by age hence, programming for AGYW should consider age-specific needs and priorities while designing interventions. In terms of HIV prevention, condom availability and accessibility should increase, and condom negotiation and use especially with non marital non cohabiting partners should be prioritized. Programmes should also scale up demand generation and access to PrEP among AGYW. Most countries had a high self-reported HIV prevalence among AGYW but low linkage to treatment. With the latest evidence around U = U [42] the programmes must also ensure that all AGYW living with HIV are linked to ART and continue in treatment to ensure high viral suppression. Along with HIV-related services, the need for sexual and reproductive health services is a priority. To address IPV and gender-based discrimination within families and communities, integrating violence prevention and redressal mechanisms within an AGYW programme is critical. Addressing gender norms and inequality within families, communities, schools, and health facilities is essential to providing stigma-free services. Access to education and other skill-based training should also be a necessary part of the package offered by AGYW programmes. Advocacy with the national government for supportive policies and laws can enable a successful AGYW programme.

To conclude, the findings of this study has a number of important implications for the Global Fund supported programmes to support AGYWs sexual and reproductive health and rights. Such outcome assessments that are rapid and use simple tools should be conducted annually to understand the gaps and opportunities in programmes with AGYW for continuous improvement in coverage of the programme.

## Supporting information

S1 Appendix Country tables. (XLSX)

S1 File (DOCX)
